# *N*-[4-(Phenyl­imino­meth­yl)phen­yl]acetamide 0.67-hydrate

**DOI:** 10.1107/S1600536810039656

**Published:** 2010-10-09

**Authors:** Tariq Mahmud, Khalid H. Thebo, Rabia Rehman, Mohammad A. Malik, Madeleine Helliwell

**Affiliations:** a Institute of Chemistry, University of the Punjab, Lahore 54590, Pakistan; b School of Chemistry and School of Materials, The University of Manchester, Oxford Road, Manchester M13 9PL, England

## Abstract

The title compound, C_15_H_14_N_2_O·0.67H_2_O, was prepared by the reaction of 4-acetoamine­benzaldehyde and aniline. The asymmetric unit contains six organic mol­ecules and four water mol­ecules. The dihedral angles between the aromatic ring planes in each organic mol­ecule vary from 42.4 (2) to 53.8 (2)°. In the crystal, an extensive network of inter­molecular N—H⋯O, O—H⋯N and O—H⋯O hydrogen bonds link the mol­ecules into [010] chains.

## Related literature

For background to polydentate Schiff bases in coordination chemistry, see: Souza *et al.* (1985 ▶); Dixit *et al.* (2009 ▶). For information on their uses as stereospecific catalysts, see: Kureshy *et al.* (1999 ▶); Aoyama *et al.* (1986 ▶).
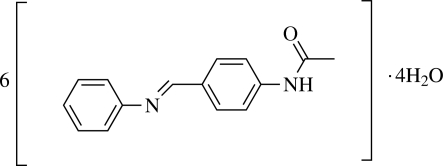


## Experimental

### 

#### Crystal data

C_15_H_14_N_2_O·0.67H_2_O
*M* *_r_* = 250.29
Monoclinic, 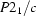

*a* = 21.328 (4) Å
*b* = 17.797 (3) Å
*c* = 23.021 (4) Å
β = 117.244 (4)°
*V* = 7769 (3) Å^3^
*Z* = 24
Mo *K*α radiation
μ = 0.09 mm^−1^
*T* = 100 K
0.60 × 0.35 × 0.10 mm

#### Data collection

Bruker SMART CCD diffractometer
40121 measured reflections
13696 independent reflections
4483 reflections with *I* > 2σ(*I*)
*R*_int_ = 0.113

#### Refinement

*R*[*F* ^2^ > 2σ(*F* ^2^)] = 0.055
*wR*(*F* ^2^) = 0.119
*S* = 0.76
13696 reflections
1057 parameters
23 restraints
H atoms treated by a mixture of independent and constrained refinement
Δρ_max_ = 0.34 e Å^−3^
Δρ_min_ = −0.21 e Å^−3^

### 

Data collection: *SMART* (Bruker, 2001 ▶); cell refinement: *SAINT* (Bruker, 2002 ▶); data reduction: *SAINT*; program(s) used to solve structure: *SHELXS97* (Sheldrick, 2008 ▶); program(s) used to refine structure: *SHELXL97* (Sheldrick, 2008 ▶); molecular graphics: *SHELXTL* (Sheldrick, 2008 ▶) and *PLATON* (Spek, 2009 ▶); software used to prepare material for publication: *SHELXTL* and *PLATON*.

## Supplementary Material

Crystal structure: contains datablocks global, I. DOI: 10.1107/S1600536810039656/hb5642sup1.cif

Structure factors: contains datablocks I. DOI: 10.1107/S1600536810039656/hb5642Isup2.hkl

Additional supplementary materials:  crystallographic information; 3D view; checkCIF report

## Figures and Tables

**Table 1:** Hydrogen-bond geometry (Å, °)

*D*—H⋯*A*	*D*—H	H⋯*A*	*D*⋯*A*	*D*—H⋯*A*
N1—H1*N*⋯O1*S*	0.88 (2)	2.00 (2)	2.872 (5)	173 (4)
N3—H3*N*⋯O2*S*	0.87 (2)	1.99 (2)	2.859 (5)	173 (4)
N5—H5*N*⋯O4*S*	0.86 (2)	2.09 (2)	2.919 (5)	163 (4)
N7—H7*N*⋯O3*S*	0.86 (2)	2.03 (2)	2.858 (5)	161 (4)
N9—H9*N*⋯O4	0.87 (2)	2.10 (2)	2.929 (5)	158 (4)
N11—H11*N*⋯O2	0.87 (2)	2.16 (3)	2.947 (5)	151 (4)
O1*S*—H1*O*⋯N4	0.83 (2)	2.29 (3)	2.939 (5)	136 (4)
O1*S*—H2*O*⋯O5	0.88 (2)	1.88 (2)	2.764 (5)	173 (4)
O2*S*—H3*O*⋯N2	0.85 (2)	2.16 (2)	3.006 (5)	177 (4)
O2*S*—H4*O*⋯O3	0.86 (2)	1.96 (2)	2.781 (5)	160 (5)
O3*S*—H5*O*⋯N6^i^	0.85 (2)	2.33 (3)	3.001 (5)	136 (3)
O3*S*—H6*O*⋯O1^i^	0.89 (2)	1.90 (2)	2.778 (4)	172 (4)
O4*S*—H7*O*⋯N8^ii^	0.87 (2)	2.09 (2)	2.954 (5)	171 (4)
O4*S*—H8*O*⋯O6^ii^	0.98 (2)	1.76 (2)	2.726 (5)	170 (4)

Symmetry codes: (i) 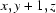; (ii) 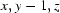.

**Fig. 1.**
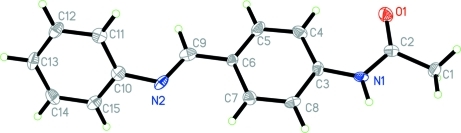
Plot of one molecule of (I) with ellipsoids drawn at the 50% probability level.

**Fig. 2.**
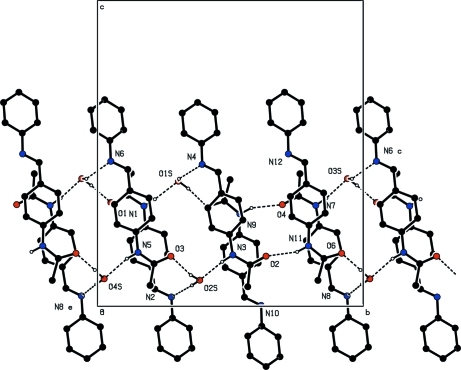
Plot showing the packing arrangement of the crystal structure of (I) viewed down a showing pairs of molecules linked to each other by hydrogen bonding interactions, to form a chain along b. Only H atoms involved in hydrogen bonding are shown. Symmetry codes: (c) x, y+1, z; (e) x, y-1, z.

## supplementary crystallographic information

### Comment

Polydentate Schiff base ligands are widely used in the preparation of transition metal complexes (Souza *et al.*, 1985). Some such complexes possess binding sites and cavities for various cations, anions and organic molecules (Dixit *et al.*, 2009). In addition, Schiff base metal complexes can be effective as stereospecific catalysts for oxidation (Kureshy *et al.*, 1999), reduction (Aoyama *et al.*, 1986) and other transformations in organic and inorganic chemistry. The title Schiff base compound was synthesized from 4-acetoaminebenzaldehyde and aniline as a potential ligand for the preparation of transition metal complexes.

The asymmetric unit contains six molecules of 4-acetylaminobenzylidine aniline (I) and four water molecules. Figure 1 shows a plot of one of the molecules of (I). The dihedral angles between the two aromatic ring planes in each molecule of (I) vary from 42.4 (2) to 53.8 (2)° (Table 2). The six molecules in the asymetric unit are arranged in pairs, one above the other. There are possible π-stacking interactions between the carbonyl group of each molecule in the pair with the central phenyl group of the molecule above or below it, with carbonyl O and C atoms to aromatic ring distances varying from 3.002 (4) to 3.421 (4) Å and 3.191 (6) to 3.492 (6) Å, respectively (Table 3, Figure 2). The paired molecules are further linked to one another by O—H···N and O—H···O hydrogen bonds from two water molecules, one at each side of the pair (Table 1, Figure 2). Each pair of molecules is linked to adjacent pairs on either side by N—H···O contacts (Table 3), either to water O atoms (*i.e.* the N1—H1···O1S, N3—H3···O2S, N5—H5···O4S and N7—H7···O3S contacts) or to a carbonyl oxygen of the next molecule (*i.e.* the N11—H11···O2 and the N9—H9···O4 contacts). In this way, the pairs of molecules are linked into chains along b, as shown in Figure 2.

### Experimental

0.0163 g (1.0 mmol) of 4-acetoaminebenzaldehyde and 0.0911 ml (1.0 mmol) of aniline were dissolved in 10.0 ml 95% ethanol separately. Both solutions were mixed in 100 ml round bottom flask. The mixture was refluxed for two hours at 423 K. It was then recrystallized by using a mixture of acetonitrile, methanol and water in the ratio 1: 3: 4 respectively.

### Refinement

H atoms bonded to C were included in calculated positions using the riding method, with aromatic and methyl C—H distances of 0.98 and 0.95 Å, respectively and U_eq_ values 1.2 and 1.5 times those of the parent atoms; the torsion angles of the methyl H atoms were optimized to give the best fit to the electron density. H atoms bonded to N and O were found by difference Fourier methods and refined isotropically with restraints on the bond lengths and with the *U*_eq_ value constrained to be 1.2 or 1.5 times that of the parent atom. The N—H distances vary from 0.86 (2) to 0.88 (2) Å and the O—H distances range from 0.83 (2) to 0.98 (2) Å.

### Crystal data

**Table:**

C_15_H_14_N_2_O·0.67H_2_O	*F*(000) = 3184
*M**_r_* = 250.29	*D*_x_ = 1.284 Mg m^−^^3^
Monoclinic, *P*2_1_/*c*	Mo *K*α radiation, λ = 0.71073 Å
Hall symbol: -P 2ybc	Cell parameters from 766 reflections
*a* = 21.328 (4) Å	θ = 2.9–24.5°
*b* = 17.797 (3) Å	µ = 0.09 mm^−^^1^
*c* = 23.021 (4) Å	*T* = 100 K
β = 117.244 (4)°	Plate, colourless
*V* = 7769 (3) Å^3^	0.60 × 0.35 × 0.10 mm
*Z* = 24	

### Data collection

**Table:**

Bruker SMART CCD diffractometer	4483 reflections with *I* > 2σ(*I*)
Radiation source: fine-focus sealed tube	*R*_int_ = 0.113
graphite	θ_max_ = 25.0°, θ_min_ = 2.1°
phi and ω scans	*h* = −25→24
40121 measured reflections	*k* = −20→21
13696 independent reflections	*l* = −27→26

### Refinement

**Table:**

Refinement on *F*^2^	Primary atom site location: structure-invariant direct methods
Least-squares matrix: full	Secondary atom site location: difference Fourier map
*R*[*F*^2^ > 2σ(*F*^2^)] = 0.055	Hydrogen site location: inferred from neighbouring sites
*wR*(*F*^2^) = 0.119	H atoms treated by a mixture of independent and constrained refinement
*S* = 0.76	*w* = 1/[σ^2^(*F*_o_^2^) + (0.0147*P*)^2^] where *P* = (*F*_o_^2^ + 2*F*_c_^2^)/3
13696 reflections	(Δ/σ)_max_ = 0.001
1057 parameters	Δρ_max_ = 0.34 e Å^−^^3^
23 restraints	Δρ_min_ = −0.21 e Å^−^^3^

### Special details

**Table:**

Geometry. All esds (except the esd in the dihedral angle between two l.s. planes) are estimated using the full covariance matrix. The cell esds are taken into account individually in the estimation of esds in distances, angles and torsion angles; correlations between esds in cell parameters are only used when they are defined by crystal symmetry. An approximate (isotropic) treatment of cell esds is used for estimating esds involving l.s. planes.
Refinement. Refinement of *F*^2^ against ALL reflections. The weighted *R*-factor wR and goodness of fit *S* are based on *F*^2^, conventional *R*-factors *R* are based on *F*, with *F* set to zero for negative *F*^2^. The threshold expression of *F*^2^ > σ(*F*^2^) is used only for calculating *R*-factors(gt) etc. and is not relevant to the choice of reflections for refinement. *R*-factors based on *F*^2^ are statistically about twice as large as those based on *F*, and *R*- factors based on ALL data will be even larger.

### Fractional atomic coordinates and isotropic or equivalent isotropic displacement parameters (Å^2^)

**Table:**

	*x*	*y*	*z*	*U*_iso_*/*U*_eq_	
O1	0.18362 (17)	0.05033 (18)	0.33952 (15)	0.0264 (9)	
N1	0.1999 (2)	0.1750 (2)	0.3278 (2)	0.0174 (10)	
H1N	0.2190 (19)	0.2159 (14)	0.3501 (16)	0.021*	
N2	0.1041 (2)	0.2783 (2)	0.03041 (18)	0.0190 (11)	
C1	0.2263 (2)	0.1271 (2)	0.43504 (19)	0.0220 (12)	
H1A	0.1854	0.1353	0.4429	0.033*	
H1B	0.2568	0.1716	0.4489	0.033*	
H1C	0.2528	0.0833	0.4600	0.033*	
C2	0.2016 (2)	0.1134 (3)	0.3628 (2)	0.0169 (12)	
C3	0.1748 (2)	0.1801 (3)	0.2598 (2)	0.0175 (12)	
C4	0.1439 (2)	0.1227 (3)	0.2154 (2)	0.0221 (13)	
H4	0.1389	0.0742	0.2301	0.027*	
C5	0.1203 (2)	0.1357 (3)	0.1500 (2)	0.0239 (13)	
H5	0.0995	0.0955	0.1201	0.029*	
C6	0.1259 (2)	0.2050 (3)	0.1263 (2)	0.0151 (12)	
C7	0.1558 (2)	0.2640 (3)	0.1704 (2)	0.0212 (13)	
H7	0.1602	0.3124	0.1552	0.025*	
C8	0.1792 (2)	0.2516 (3)	0.2364 (2)	0.0201 (12)	
H8	0.1985	0.2921	0.2662	0.024*	
C9	0.1008 (3)	0.2157 (3)	0.0562 (2)	0.0221 (13)	
H9	0.0809	0.1738	0.0282	0.027*	
C10	0.0809 (3)	0.2823 (3)	−0.0384 (2)	0.0203 (13)	
C11	0.0973 (2)	0.2264 (3)	−0.0729 (2)	0.0203 (12)	
H11	0.1219	0.1822	−0.0512	0.024*	
C12	0.0771 (2)	0.2368 (3)	−0.1387 (2)	0.0235 (13)	
H12	0.0881	0.1994	−0.1621	0.028*	
C13	0.0411 (3)	0.3008 (3)	−0.1708 (2)	0.0245 (14)	
H13	0.0280	0.3075	−0.2159	0.029*	
C14	0.0244 (3)	0.3552 (3)	−0.1371 (2)	0.0244 (14)	
H14	−0.0010	0.3988	−0.1593	0.029*	
C15	0.0447 (3)	0.3462 (3)	−0.0713 (2)	0.0213 (13)	
H15	0.0338	0.3841	−0.0483	0.026*	
O2	0.31874 (18)	0.63435 (18)	0.16385 (15)	0.0252 (9)	
N3	0.2971 (2)	0.5096 (2)	0.16793 (19)	0.0168 (10)	
H3N	0.2778 (19)	0.4694 (15)	0.1456 (17)	0.020*	
N4	0.3898 (2)	0.3942 (2)	0.46400 (18)	0.0209 (11)	
C16	0.2677 (2)	0.5655 (3)	0.0640 (2)	0.0287 (13)	
H16A	0.2327	0.6048	0.0415	0.043*	
H16B	0.2454	0.5161	0.0508	0.043*	
H16C	0.3063	0.5697	0.0522	0.043*	
C17	0.2961 (2)	0.5746 (3)	0.1357 (2)	0.0203 (12)	
C18	0.3253 (2)	0.4967 (3)	0.2350 (2)	0.0182 (12)	
C19	0.3067 (2)	0.4306 (3)	0.2562 (2)	0.0196 (12)	
H19	0.2764	0.3954	0.2249	0.024*	
C20	0.3318 (2)	0.4156 (3)	0.3219 (2)	0.0183 (12)	
H20	0.3191	0.3699	0.3351	0.022*	
C21	0.3753 (2)	0.4664 (3)	0.3694 (2)	0.0192 (13)	
C22	0.3943 (2)	0.5310 (3)	0.3481 (2)	0.0209 (13)	
H22	0.4247	0.5658	0.3798	0.025*	
C23	0.3710 (2)	0.5473 (3)	0.2821 (2)	0.0203 (12)	
H23	0.3857	0.5920	0.2693	0.024*	
C24	0.4008 (2)	0.4543 (3)	0.4389 (2)	0.0210 (13)	
H24	0.4274	0.4933	0.4678	0.025*	
C25	0.4151 (3)	0.3917 (3)	0.5331 (2)	0.0202 (13)	
C26	0.4487 (3)	0.3259 (3)	0.5653 (2)	0.0233 (13)	
H26	0.4567	0.2862	0.5419	0.028*	
C27	0.4705 (3)	0.3189 (3)	0.6320 (3)	0.0276 (14)	
H27	0.4939	0.2745	0.6542	0.033*	
C28	0.4581 (2)	0.3768 (3)	0.6666 (2)	0.0268 (14)	
H28	0.4728	0.3716	0.7121	0.032*	
C29	0.4245 (2)	0.4418 (3)	0.6343 (2)	0.0273 (14)	
H29	0.4163	0.4812	0.6579	0.033*	
C30	0.4025 (2)	0.4500 (3)	0.5670 (2)	0.0241 (13)	
H30	0.3792	0.4945	0.5449	0.029*	
O3	0.32175 (18)	0.27632 (18)	0.16364 (15)	0.0270 (9)	
N5	0.3035 (2)	0.1528 (2)	0.1761 (2)	0.0240 (11)	
H5N	0.293 (2)	0.1107 (14)	0.1558 (18)	0.029*	
N6	0.3977 (2)	0.0503 (2)	0.47430 (19)	0.0223 (11)	
C31	0.2754 (2)	0.1980 (2)	0.0682 (2)	0.0255 (13)	
H31A	0.2408	0.2364	0.0428	0.038*	
H31B	0.2531	0.1483	0.0575	0.038*	
H31C	0.3149	0.1993	0.0574	0.038*	
C32	0.3021 (3)	0.2130 (3)	0.1394 (2)	0.0251 (13)	
C33	0.3298 (2)	0.1451 (3)	0.2438 (2)	0.0178 (12)	
C34	0.3252 (2)	0.0740 (3)	0.2680 (2)	0.0241 (13)	
H34	0.3065	0.0327	0.2388	0.029*	
C35	0.3475 (2)	0.0640 (3)	0.3333 (2)	0.0204 (13)	
H35	0.3442	0.0154	0.3487	0.025*	
C36	0.3749 (2)	0.1224 (3)	0.3781 (2)	0.0182 (12)	
C37	0.3796 (2)	0.1938 (3)	0.3543 (2)	0.0227 (13)	
H37	0.3982	0.2348	0.3837	0.027*	
C38	0.3571 (2)	0.2049 (3)	0.2877 (2)	0.0229 (13)	
H38	0.3604	0.2534	0.2721	0.028*	
C39	0.3986 (2)	0.1140 (3)	0.4476 (2)	0.0220 (13)	
H39	0.4154	0.1571	0.4748	0.026*	
C40	0.4198 (2)	0.0468 (3)	0.5425 (2)	0.0184 (12)	
C41	0.4593 (2)	−0.0158 (3)	0.5761 (2)	0.0224 (13)	
H41	0.4716	−0.0527	0.5532	0.027*	
C42	0.4807 (3)	−0.0249 (3)	0.6421 (2)	0.0233 (13)	
H42	0.5087	−0.0671	0.6645	0.028*	
C43	0.4617 (2)	0.0272 (3)	0.6757 (2)	0.0244 (13)	
H43	0.4753	0.0205	0.7208	0.029*	
C44	0.4223 (2)	0.0896 (3)	0.6427 (2)	0.0237 (13)	
H44	0.4097	0.1261	0.6656	0.028*	
C45	0.4015 (2)	0.0987 (3)	0.5769 (2)	0.0225 (12)	
H45	0.3741	0.1414	0.5548	0.027*	
O4	0.17838 (17)	0.69595 (16)	0.33253 (15)	0.0282 (8)	
N7	0.2032 (2)	0.8216 (2)	0.3298 (2)	0.0213 (11)	
H7N	0.216 (2)	0.8614 (16)	0.3537 (17)	0.026*	
N8	0.1046 (2)	0.9396 (2)	0.03385 (18)	0.0231 (10)	
C46	0.2345 (2)	0.7620 (2)	0.43357 (19)	0.0301 (13)	
H46A	0.1969	0.7642	0.4468	0.045*	
H46B	0.2638	0.8073	0.4488	0.045*	
H46C	0.2638	0.7175	0.4529	0.045*	
C47	0.2022 (2)	0.7574 (3)	0.3598 (2)	0.0212 (12)	
C48	0.1736 (2)	0.8347 (2)	0.2619 (2)	0.0154 (11)	
C49	0.1905 (2)	0.9016 (2)	0.2411 (2)	0.0194 (11)	
H49	0.2206	0.9364	0.2729	0.023*	
C50	0.1653 (2)	0.9189 (3)	0.1764 (2)	0.0211 (12)	
H50	0.1779	0.9650	0.1637	0.025*	
C51	0.1202 (2)	0.8678 (3)	0.1287 (2)	0.0211 (13)	
C52	0.1005 (3)	0.8029 (3)	0.1492 (2)	0.0235 (13)	
H52	0.0685	0.7692	0.1175	0.028*	
C53	0.1264 (2)	0.7862 (3)	0.2149 (2)	0.0241 (13)	
H53	0.1119	0.7415	0.2278	0.029*	
C54	0.0920 (3)	0.8812 (3)	0.0589 (2)	0.0255 (13)	
H54	0.0620	0.8439	0.0300	0.031*	
C55	0.0762 (3)	0.9445 (3)	−0.0355 (2)	0.0214 (13)	
C56	0.0460 (2)	1.0108 (3)	−0.0670 (2)	0.0284 (14)	
H56	0.0433	1.0518	−0.0419	0.034*	
C57	0.0195 (3)	1.0197 (3)	−0.1337 (2)	0.0290 (14)	
H57	−0.0021	1.0656	−0.1540	0.035*	
C58	0.0249 (2)	0.9607 (3)	−0.1710 (2)	0.0277 (13)	
H58	0.0077	0.9661	−0.2168	0.033*	
C59	0.0555 (2)	0.8945 (3)	−0.1400 (2)	0.0279 (14)	
H59	0.0600	0.8544	−0.1651	0.034*	
C60	0.0800 (2)	0.8846 (3)	−0.0738 (2)	0.0235 (12)	
H60	0.0993	0.8376	−0.0540	0.028*	
O5	0.17220 (17)	0.41264 (18)	0.32528 (15)	0.0268 (9)	
N9	0.1768 (2)	0.5360 (2)	0.30192 (19)	0.0212 (10)	
H9N	0.186 (2)	0.5795 (13)	0.3216 (17)	0.025*	
N10	0.0890 (2)	0.6160 (2)	0.0028 (2)	0.0278 (11)	
C61	0.2153 (2)	0.4991 (2)	0.4143 (2)	0.0312 (14)	
H61A	0.1866	0.4763	0.4329	0.047*	
H61B	0.2144	0.5539	0.4181	0.047*	
H61C	0.2639	0.4811	0.4382	0.047*	
C62	0.1860 (3)	0.4777 (3)	0.3438 (2)	0.0225 (13)	
C63	0.1546 (2)	0.5338 (3)	0.2337 (2)	0.0234 (13)	
C64	0.1207 (2)	0.4740 (3)	0.1929 (2)	0.0242 (13)	
H64	0.1115	0.4291	0.2100	0.029*	
C65	0.1004 (2)	0.4807 (3)	0.1266 (2)	0.0255 (13)	
H65	0.0781	0.4391	0.0989	0.031*	
C66	0.1113 (2)	0.5452 (3)	0.0990 (2)	0.0219 (13)	
C67	0.1455 (2)	0.6046 (3)	0.1412 (2)	0.0248 (13)	
H67	0.1541	0.6497	0.1240	0.030*	
C68	0.1668 (2)	0.5993 (3)	0.2069 (2)	0.0206 (13)	
H68	0.1902	0.6406	0.2346	0.025*	
C69	0.0883 (2)	0.5532 (3)	0.0296 (2)	0.0252 (13)	
H69	0.0720	0.5098	0.0027	0.030*	
C70	0.0689 (3)	0.6184 (3)	−0.0650 (2)	0.0228 (13)	
C71	0.0906 (2)	0.5658 (3)	−0.0972 (2)	0.0278 (13)	
H71	0.1185	0.5240	−0.0739	0.033*	
C72	0.0718 (3)	0.5742 (3)	−0.1622 (2)	0.0303 (13)	
H72	0.0873	0.5386	−0.1836	0.036*	
C73	0.0302 (3)	0.6346 (3)	−0.1970 (2)	0.0300 (14)	
H73	0.0166	0.6400	−0.2422	0.036*	
C74	0.0087 (3)	0.6869 (3)	−0.1652 (2)	0.0321 (14)	
H74	−0.0201	0.7280	−0.1888	0.039*	
C75	0.0287 (3)	0.6797 (3)	−0.0999 (2)	0.0250 (14)	
H75	0.0151	0.7167	−0.0782	0.030*	
O6	0.32500 (17)	0.91894 (19)	0.17329 (16)	0.0318 (9)	
N11	0.3190 (2)	0.7942 (2)	0.1974 (2)	0.0313 (11)	
H11N	0.303 (2)	0.7515 (15)	0.1780 (19)	0.038*	
N12	0.4177 (2)	0.7185 (2)	0.4983 (2)	0.0247 (11)	
C76	0.2809 (2)	0.8298 (2)	0.0847 (2)	0.0339 (13)	
H76A	0.3195	0.8270	0.0729	0.051*	
H76B	0.2463	0.8672	0.0573	0.051*	
H76C	0.2580	0.7806	0.0781	0.051*	
C77	0.3103 (3)	0.8524 (3)	0.1566 (2)	0.0237 (13)	
C78	0.3446 (2)	0.7961 (3)	0.2651 (2)	0.0218 (12)	
C79	0.3814 (2)	0.8570 (3)	0.3046 (2)	0.0268 (14)	
H79	0.3905	0.9010	0.2862	0.032*	
C80	0.4041 (3)	0.8512 (3)	0.3707 (2)	0.0250 (13)	
H80	0.4277	0.8928	0.3977	0.030*	
C81	0.3938 (2)	0.7863 (3)	0.4002 (2)	0.0227 (13)	
C82	0.3566 (2)	0.7262 (3)	0.3592 (2)	0.0260 (13)	
H82	0.3482	0.6818	0.3774	0.031*	
C83	0.3323 (2)	0.7318 (3)	0.2930 (2)	0.0272 (13)	
H83	0.3067	0.6912	0.2657	0.033*	
C84	0.4202 (2)	0.7804 (3)	0.4706 (2)	0.0277 (14)	
H84	0.4399	0.8236	0.4969	0.033*	
C85	0.4417 (3)	0.7169 (3)	0.5663 (2)	0.0263 (13)	
C86	0.4285 (2)	0.7739 (3)	0.6005 (2)	0.0284 (14)	
H86	0.4042	0.8179	0.5780	0.034*	
C87	0.4501 (2)	0.7675 (3)	0.6665 (2)	0.0311 (14)	
H87	0.4403	0.8067	0.6892	0.037*	
C88	0.4860 (3)	0.7042 (3)	0.7001 (2)	0.0324 (14)	
H88	0.5016	0.7003	0.7458	0.039*	
C89	0.4991 (3)	0.6464 (3)	0.6666 (2)	0.0321 (14)	
H89	0.5238	0.6028	0.6894	0.039*	
C90	0.4764 (3)	0.6522 (3)	0.6004 (2)	0.0287 (14)	
H90	0.4843	0.6117	0.5776	0.034*	
O1S	0.25984 (18)	0.30429 (19)	0.40922 (16)	0.0264 (9)	
H1O	0.3019 (11)	0.307 (2)	0.417 (2)	0.040*	
H2O	0.2331 (19)	0.3415 (17)	0.3852 (18)	0.040*	
O2S	0.22697 (18)	0.38491 (18)	0.08542 (16)	0.0281 (9)	
H3O	0.1920 (16)	0.356 (2)	0.0685 (18)	0.042*	
H4O	0.2586 (19)	0.351 (2)	0.102 (2)	0.042*	
O3S	0.27737 (17)	0.94103 (17)	0.41746 (15)	0.0292 (8)	
H5O	0.3144 (15)	0.958 (2)	0.417 (2)	0.044*	
H6O	0.2460 (18)	0.9762 (19)	0.3956 (19)	0.044*	
O4S	0.23815 (18)	0.02510 (18)	0.08989 (16)	0.0360 (9)	
H7O	0.2016 (16)	−0.004 (2)	0.0729 (19)	0.054*	
H8O	0.2736 (18)	−0.010 (2)	0.1188 (18)	0.054*	

### Atomic displacement parameters (Å^2^)

**Table:**

	*U*^11^	*U*^22^	*U*^33^	*U*^12^	*U*^13^	*U*^23^
O1	0.030 (2)	0.021 (2)	0.024 (2)	−0.0057 (17)	0.0088 (16)	0.0027 (17)
N1	0.018 (2)	0.010 (2)	0.023 (3)	−0.0049 (19)	0.0082 (19)	−0.0028 (19)
N2	0.015 (2)	0.027 (3)	0.016 (3)	−0.007 (2)	0.0076 (19)	−0.010 (2)
C1	0.024 (3)	0.024 (3)	0.017 (3)	−0.002 (2)	0.008 (2)	−0.003 (2)
C2	0.010 (3)	0.016 (3)	0.025 (3)	0.001 (2)	0.008 (2)	−0.002 (2)
C3	0.016 (3)	0.022 (3)	0.015 (3)	0.007 (2)	0.007 (2)	0.003 (2)
C4	0.021 (3)	0.016 (3)	0.027 (3)	−0.003 (2)	0.009 (2)	0.002 (2)
C5	0.024 (3)	0.027 (3)	0.016 (3)	−0.007 (2)	0.006 (2)	−0.007 (2)
C6	0.013 (3)	0.016 (3)	0.012 (3)	0.001 (2)	0.002 (2)	−0.002 (2)
C7	0.024 (3)	0.021 (3)	0.022 (3)	−0.001 (2)	0.012 (2)	0.001 (2)
C8	0.017 (3)	0.017 (3)	0.024 (3)	−0.003 (2)	0.008 (2)	−0.008 (2)
C9	0.016 (3)	0.021 (3)	0.029 (3)	−0.003 (3)	0.011 (3)	−0.007 (3)
C10	0.016 (3)	0.024 (3)	0.024 (3)	−0.007 (2)	0.012 (2)	−0.003 (3)
C11	0.013 (3)	0.024 (3)	0.023 (3)	0.002 (2)	0.006 (2)	0.001 (2)
C12	0.020 (3)	0.031 (3)	0.017 (3)	−0.006 (2)	0.007 (2)	−0.009 (2)
C13	0.024 (3)	0.036 (4)	0.013 (3)	−0.004 (3)	0.008 (2)	−0.002 (3)
C14	0.022 (3)	0.024 (3)	0.023 (3)	−0.002 (3)	0.005 (2)	0.007 (3)
C15	0.018 (3)	0.025 (3)	0.021 (3)	−0.002 (3)	0.009 (2)	−0.006 (3)
O2	0.033 (2)	0.018 (2)	0.025 (2)	−0.0072 (17)	0.0134 (16)	−0.0051 (17)
N3	0.021 (3)	0.013 (2)	0.011 (2)	−0.0001 (19)	0.0037 (19)	−0.0033 (19)
N4	0.022 (3)	0.022 (3)	0.018 (3)	0.003 (2)	0.009 (2)	0.006 (2)
C16	0.029 (3)	0.028 (3)	0.031 (3)	−0.001 (2)	0.016 (3)	0.004 (3)
C17	0.018 (3)	0.024 (3)	0.021 (3)	0.001 (2)	0.011 (2)	0.006 (3)
C18	0.018 (3)	0.020 (3)	0.023 (3)	−0.004 (2)	0.014 (2)	−0.006 (2)
C19	0.015 (3)	0.024 (3)	0.018 (3)	−0.004 (2)	0.006 (2)	−0.005 (2)
C20	0.024 (3)	0.008 (3)	0.027 (3)	−0.002 (2)	0.016 (2)	−0.003 (2)
C21	0.015 (3)	0.019 (3)	0.027 (3)	0.002 (2)	0.012 (2)	0.004 (3)
C22	0.021 (3)	0.014 (3)	0.019 (3)	−0.002 (2)	0.002 (2)	−0.004 (2)
C23	0.024 (3)	0.016 (3)	0.020 (3)	−0.001 (2)	0.010 (2)	0.001 (2)
C24	0.012 (3)	0.026 (3)	0.023 (3)	−0.002 (2)	0.007 (2)	−0.007 (3)
C25	0.015 (3)	0.021 (3)	0.021 (3)	−0.006 (2)	0.006 (2)	0.000 (3)
C26	0.024 (3)	0.023 (3)	0.024 (3)	−0.007 (3)	0.011 (2)	−0.003 (3)
C27	0.021 (3)	0.027 (3)	0.034 (4)	−0.003 (3)	0.011 (3)	0.006 (3)
C28	0.017 (3)	0.043 (4)	0.017 (3)	−0.002 (3)	0.005 (2)	0.006 (3)
C29	0.023 (3)	0.032 (3)	0.025 (3)	−0.004 (2)	0.010 (2)	−0.007 (3)
C30	0.023 (3)	0.024 (3)	0.022 (3)	−0.006 (2)	0.008 (2)	−0.004 (2)
O3	0.031 (2)	0.019 (2)	0.027 (2)	−0.0077 (17)	0.0102 (16)	−0.0071 (17)
N5	0.028 (3)	0.021 (3)	0.025 (3)	0.000 (2)	0.013 (2)	−0.006 (2)
N6	0.021 (2)	0.019 (3)	0.027 (3)	0.0032 (19)	0.0101 (19)	0.003 (2)
C31	0.021 (3)	0.029 (3)	0.029 (3)	−0.005 (2)	0.013 (2)	−0.001 (2)
C32	0.018 (3)	0.032 (4)	0.029 (3)	0.000 (3)	0.013 (2)	0.002 (3)
C33	0.015 (3)	0.016 (3)	0.025 (3)	−0.003 (2)	0.010 (2)	−0.006 (2)
C34	0.026 (3)	0.024 (3)	0.023 (3)	0.000 (2)	0.011 (2)	0.002 (2)
C35	0.020 (3)	0.014 (3)	0.031 (3)	0.001 (2)	0.015 (2)	−0.003 (3)
C36	0.015 (3)	0.022 (3)	0.021 (3)	0.000 (2)	0.011 (2)	0.004 (2)
C37	0.016 (3)	0.020 (3)	0.032 (3)	−0.001 (2)	0.011 (2)	−0.005 (2)
C38	0.025 (3)	0.016 (3)	0.034 (3)	0.001 (2)	0.019 (3)	−0.001 (2)
C39	0.016 (3)	0.018 (3)	0.033 (3)	−0.001 (2)	0.012 (2)	−0.006 (3)
C40	0.012 (3)	0.019 (3)	0.020 (3)	−0.002 (2)	0.004 (2)	0.004 (2)
C41	0.021 (3)	0.020 (3)	0.028 (3)	−0.006 (2)	0.013 (2)	−0.004 (2)
C42	0.021 (3)	0.023 (3)	0.031 (3)	0.004 (2)	0.016 (3)	0.001 (3)
C43	0.017 (3)	0.032 (3)	0.022 (3)	−0.002 (3)	0.007 (2)	0.000 (3)
C44	0.017 (3)	0.029 (3)	0.026 (3)	−0.004 (2)	0.011 (2)	−0.013 (3)
C45	0.021 (3)	0.017 (3)	0.029 (3)	−0.004 (2)	0.011 (2)	−0.002 (2)
O4	0.037 (2)	0.0192 (19)	0.029 (2)	−0.0047 (17)	0.0163 (17)	−0.0014 (17)
N7	0.023 (3)	0.017 (3)	0.024 (3)	−0.006 (2)	0.011 (2)	−0.005 (2)
N8	0.021 (2)	0.025 (3)	0.021 (3)	−0.001 (2)	0.0082 (19)	−0.001 (2)
C46	0.029 (3)	0.034 (3)	0.025 (3)	−0.006 (3)	0.011 (2)	0.001 (3)
C47	0.022 (3)	0.018 (3)	0.031 (3)	−0.006 (2)	0.018 (2)	−0.010 (3)
C48	0.014 (3)	0.013 (3)	0.018 (3)	0.004 (2)	0.006 (2)	0.002 (2)
C49	0.028 (3)	0.012 (3)	0.019 (3)	−0.004 (2)	0.011 (2)	−0.004 (2)
C50	0.017 (3)	0.021 (3)	0.025 (3)	−0.007 (2)	0.009 (2)	−0.005 (2)
C51	0.020 (3)	0.028 (3)	0.015 (3)	0.003 (2)	0.008 (2)	−0.003 (2)
C52	0.017 (3)	0.026 (3)	0.025 (3)	−0.003 (2)	0.008 (2)	−0.004 (3)
C53	0.018 (3)	0.025 (3)	0.026 (3)	−0.008 (2)	0.006 (2)	−0.006 (2)
C54	0.017 (3)	0.019 (3)	0.039 (4)	−0.004 (2)	0.012 (3)	−0.010 (3)
C55	0.019 (3)	0.020 (3)	0.025 (3)	−0.004 (2)	0.010 (2)	−0.005 (3)
C56	0.023 (3)	0.032 (3)	0.036 (4)	0.005 (3)	0.019 (3)	0.003 (3)
C57	0.030 (3)	0.029 (3)	0.031 (4)	0.002 (3)	0.016 (3)	0.007 (3)
C58	0.026 (3)	0.035 (3)	0.025 (3)	−0.001 (2)	0.013 (2)	0.004 (3)
C59	0.021 (3)	0.036 (3)	0.028 (3)	−0.006 (2)	0.012 (2)	−0.009 (3)
C60	0.019 (3)	0.020 (3)	0.028 (3)	−0.005 (2)	0.008 (2)	0.004 (2)
O5	0.032 (2)	0.016 (2)	0.031 (2)	0.0005 (17)	0.0135 (17)	0.0008 (18)
N9	0.024 (2)	0.010 (2)	0.032 (3)	−0.001 (2)	0.015 (2)	−0.005 (2)
N10	0.031 (3)	0.022 (3)	0.036 (3)	0.005 (2)	0.021 (2)	−0.001 (2)
C61	0.030 (3)	0.029 (3)	0.033 (3)	0.002 (3)	0.013 (3)	−0.003 (3)
C62	0.016 (3)	0.028 (3)	0.027 (3)	0.002 (3)	0.012 (2)	−0.003 (3)
C63	0.016 (3)	0.030 (3)	0.028 (3)	0.000 (2)	0.013 (2)	0.001 (3)
C64	0.019 (3)	0.019 (3)	0.031 (3)	−0.004 (2)	0.008 (2)	0.003 (3)
C65	0.021 (3)	0.027 (3)	0.026 (3)	−0.002 (2)	0.008 (2)	−0.008 (3)
C66	0.014 (3)	0.021 (3)	0.030 (3)	−0.004 (2)	0.010 (2)	−0.004 (3)
C67	0.023 (3)	0.024 (3)	0.032 (3)	0.002 (2)	0.015 (2)	0.008 (3)
C68	0.017 (3)	0.015 (3)	0.036 (3)	−0.008 (2)	0.018 (3)	−0.015 (3)
C69	0.020 (3)	0.030 (3)	0.022 (3)	0.000 (2)	0.007 (2)	−0.003 (3)
C70	0.019 (3)	0.026 (3)	0.022 (3)	−0.003 (2)	0.009 (2)	−0.002 (2)
C71	0.018 (3)	0.032 (3)	0.033 (3)	0.007 (2)	0.013 (2)	−0.004 (3)
C72	0.031 (3)	0.032 (3)	0.027 (3)	0.003 (3)	0.013 (2)	0.000 (2)
C73	0.025 (3)	0.044 (4)	0.023 (3)	−0.002 (3)	0.014 (2)	0.002 (3)
C74	0.027 (3)	0.033 (3)	0.040 (4)	0.005 (3)	0.018 (3)	0.014 (3)
C75	0.030 (3)	0.017 (3)	0.035 (4)	−0.001 (2)	0.022 (3)	−0.002 (3)
O6	0.030 (2)	0.029 (2)	0.036 (2)	−0.0012 (18)	0.0143 (17)	−0.0042 (19)
N11	0.025 (3)	0.032 (3)	0.033 (3)	−0.002 (2)	0.009 (2)	−0.003 (2)
N12	0.023 (3)	0.017 (3)	0.035 (3)	−0.002 (2)	0.015 (2)	−0.003 (2)
C76	0.032 (3)	0.041 (3)	0.027 (3)	0.004 (3)	0.012 (2)	−0.005 (3)
C77	0.016 (3)	0.017 (3)	0.033 (3)	0.003 (2)	0.007 (2)	0.000 (3)
C78	0.023 (3)	0.021 (3)	0.020 (3)	0.004 (2)	0.009 (2)	−0.005 (2)
C79	0.024 (3)	0.024 (3)	0.033 (3)	0.001 (2)	0.014 (3)	−0.008 (3)
C80	0.022 (3)	0.018 (3)	0.032 (3)	−0.004 (2)	0.010 (2)	−0.008 (3)
C81	0.015 (3)	0.021 (3)	0.033 (3)	−0.002 (2)	0.012 (3)	−0.009 (3)
C82	0.022 (3)	0.023 (3)	0.038 (3)	−0.002 (2)	0.017 (3)	−0.008 (3)
C83	0.025 (3)	0.022 (3)	0.037 (4)	−0.003 (2)	0.016 (3)	0.004 (3)
C84	0.021 (3)	0.025 (3)	0.042 (4)	0.002 (2)	0.019 (3)	−0.008 (3)
C85	0.021 (3)	0.024 (3)	0.037 (3)	−0.004 (2)	0.016 (3)	−0.006 (3)
C86	0.025 (3)	0.016 (3)	0.044 (4)	0.005 (2)	0.016 (3)	0.005 (3)
C87	0.026 (3)	0.033 (3)	0.032 (3)	0.005 (2)	0.012 (2)	−0.002 (3)
C88	0.034 (3)	0.030 (3)	0.036 (3)	0.004 (3)	0.019 (3)	0.004 (3)
C89	0.034 (3)	0.028 (3)	0.040 (4)	0.004 (3)	0.022 (3)	0.005 (3)
C90	0.029 (3)	0.015 (3)	0.045 (4)	0.003 (2)	0.020 (3)	0.003 (3)
O1S	0.026 (2)	0.022 (2)	0.028 (2)	−0.0022 (17)	0.0093 (18)	0.0002 (16)
O2S	0.027 (2)	0.025 (2)	0.029 (2)	−0.0046 (16)	0.0093 (18)	−0.0038 (17)
O3S	0.031 (2)	0.023 (2)	0.034 (2)	−0.0034 (16)	0.0147 (18)	−0.0014 (16)
O4S	0.039 (2)	0.027 (2)	0.035 (2)	−0.0100 (18)	0.0113 (18)	−0.0014 (17)

### Geometric parameters (Å, °)

**Table:**

O1—C2	1.227 (5)	N8—C54	1.277 (6)
N1—C2	1.350 (6)	N8—C55	1.427 (6)
N1—C3	1.407 (6)	C46—C47	1.515 (6)
N1—H1N	0.877 (17)	C46—H46A	0.9800
N2—C9	1.279 (6)	C46—H46B	0.9800
N2—C10	1.429 (6)	C46—H46C	0.9800
C1—C2	1.517 (6)	C48—C53	1.390 (6)
C1—H1A	0.9800	C48—C49	1.392 (6)
C1—H1B	0.9800	C49—C50	1.368 (6)
C1—H1C	0.9800	C49—H49	0.9500
C3—C4	1.381 (6)	C50—C51	1.410 (6)
C3—C8	1.403 (6)	C50—H50	0.9500
C4—C5	1.370 (6)	C51—C52	1.384 (6)
C4—H4	0.9500	C51—C54	1.454 (6)
C5—C6	1.377 (6)	C52—C53	1.384 (6)
C5—H5	0.9500	C52—H52	0.9500
C6—C7	1.395 (6)	C53—H53	0.9500
C6—C9	1.461 (7)	C54—H54	0.9500
C7—C8	1.382 (6)	C55—C56	1.379 (6)
C7—H7	0.9500	C55—C60	1.410 (6)
C8—H8	0.9500	C56—C57	1.381 (6)
C9—H9	0.9500	C56—H56	0.9500
C10—C15	1.388 (6)	C57—C58	1.393 (6)
C10—C11	1.415 (6)	C57—H57	0.9500
C11—C12	1.383 (6)	C58—C59	1.378 (6)
C11—H11	0.9500	C58—H58	0.9500
C12—C13	1.385 (6)	C59—C60	1.379 (6)
C12—H12	0.9500	C59—H59	0.9500
C13—C14	1.386 (6)	C60—H60	0.9500
C13—H13	0.9500	O5—C62	1.222 (5)
C14—C15	1.382 (6)	N9—C62	1.368 (6)
C14—H14	0.9500	N9—C63	1.418 (6)
C15—H15	0.9500	N9—H9N	0.873 (17)
O2—C17	1.224 (5)	N10—C69	1.280 (6)
N3—C17	1.368 (6)	N10—C70	1.418 (6)
N3—C18	1.396 (6)	C61—C62	1.499 (6)
N3—H3N	0.868 (17)	C61—H61A	0.9800
N4—C24	1.286 (6)	C61—H61B	0.9800
N4—C25	1.427 (6)	C61—H61C	0.9800
C16—C17	1.485 (6)	C63—C64	1.385 (6)
C16—H16A	0.9800	C63—C68	1.399 (6)
C16—H16B	0.9800	C64—C65	1.390 (6)
C16—H16C	0.9800	C64—H64	0.9500
C18—C19	1.400 (6)	C65—C66	1.381 (6)
C18—C23	1.402 (6)	C65—H65	0.9500
C19—C20	1.379 (6)	C66—C67	1.393 (6)
C19—H19	0.9500	C66—C69	1.449 (6)
C20—C21	1.394 (6)	C67—C68	1.369 (6)
C20—H20	0.9500	C67—H67	0.9500
C21—C22	1.381 (6)	C68—H68	0.9500
C21—C24	1.452 (6)	C69—H69	0.9500
C22—C23	1.396 (6)	C70—C75	1.393 (6)
C22—H22	0.9500	C70—C71	1.398 (6)
C23—H23	0.9500	C71—C72	1.367 (6)
C24—H24	0.9500	C71—H71	0.9500
C25—C26	1.397 (6)	C72—C73	1.392 (6)
C25—C30	1.397 (6)	C72—H72	0.9500
C26—C27	1.391 (7)	C73—C74	1.386 (6)
C26—H26	0.9500	C73—H73	0.9500
C27—C28	1.399 (6)	C74—C75	1.367 (6)
C27—H27	0.9500	C74—H74	0.9500
C28—C29	1.384 (6)	C75—H75	0.9500
C28—H28	0.9500	O6—C77	1.240 (5)
C29—C30	1.405 (6)	N11—C77	1.353 (6)
C29—H29	0.9500	N11—C78	1.396 (6)
C30—H30	0.9500	N11—H11N	0.867 (18)
O3—C32	1.242 (5)	N12—C84	1.285 (6)
N5—C32	1.357 (6)	N12—C85	1.406 (6)
N5—C33	1.401 (6)	C76—C77	1.531 (6)
N5—H5N	0.857 (17)	C76—H76A	0.9800
N6—C39	1.294 (6)	C76—H76B	0.9800
N6—C40	1.419 (6)	C76—H76C	0.9800
C31—C32	1.493 (6)	C78—C83	1.395 (6)
C31—H31A	0.9800	C78—C79	1.402 (6)
C31—H31B	0.9800	C79—C80	1.374 (6)
C31—H31C	0.9800	C79—H79	0.9500
C33—C38	1.398 (6)	C80—C81	1.406 (6)
C33—C34	1.404 (6)	C80—H80	0.9500
C34—C35	1.364 (6)	C81—C82	1.406 (6)
C34—H34	0.9500	C81—C84	1.457 (6)
C35—C36	1.391 (6)	C82—C83	1.370 (6)
C35—H35	0.9500	C82—H82	0.9500
C36—C37	1.406 (6)	C83—H83	0.9500
C36—C39	1.449 (6)	C84—H84	0.9500
C37—C38	1.395 (6)	C85—C86	1.392 (6)
C37—H37	0.9500	C85—C90	1.399 (6)
C38—H38	0.9500	C86—C87	1.377 (6)
C39—H39	0.9500	C86—H86	0.9500
C40—C45	1.384 (6)	C87—C88	1.381 (6)
C40—C41	1.398 (6)	C87—H87	0.9500
C41—C42	1.382 (6)	C88—C89	1.389 (6)
C41—H41	0.9500	C88—H88	0.9500
C42—C43	1.383 (6)	C89—C90	1.374 (6)
C42—H42	0.9500	C89—H89	0.9500
C43—C44	1.389 (6)	C90—H90	0.9500
C43—H43	0.9500	O1S—H1O	0.832 (19)
C44—C45	1.380 (6)	O1S—H2O	0.884 (18)
C44—H44	0.9500	O2S—H3O	0.845 (18)
C45—H45	0.9500	O2S—H4O	0.857 (19)
O4—C47	1.247 (5)	O3S—H5O	0.848 (18)
N7—C47	1.341 (6)	O3S—H6O	0.886 (18)
N7—C48	1.411 (6)	O4S—H7O	0.870 (19)
N7—H7N	0.860 (18)	O4S—H8O	0.977 (19)
			
C2—N1—C3	127.5 (4)	C48—N7—H7N	115 (3)
C2—N1—H1N	117 (3)	C54—N8—C55	119.1 (4)
C3—N1—H1N	116 (3)	C47—C46—H46A	109.5
C9—N2—C10	119.5 (4)	C47—C46—H46B	109.5
C2—C1—H1A	109.5	H46A—C46—H46B	109.5
C2—C1—H1B	109.5	C47—C46—H46C	109.5
H1A—C1—H1B	109.5	H46A—C46—H46C	109.5
C2—C1—H1C	109.5	H46B—C46—H46C	109.5
H1A—C1—H1C	109.5	O4—C47—N7	126.0 (4)
H1B—C1—H1C	109.5	O4—C47—C46	118.9 (4)
O1—C2—N1	124.4 (4)	N7—C47—C46	115.1 (4)
O1—C2—C1	120.6 (4)	C53—C48—C49	118.2 (4)
N1—C2—C1	115.0 (4)	C53—C48—N7	123.9 (4)
C4—C3—C8	118.7 (4)	C49—C48—N7	117.8 (4)
C4—C3—N1	125.8 (5)	C50—C49—C48	122.2 (4)
C8—C3—N1	115.4 (5)	C50—C49—H49	118.9
C5—C4—C3	119.9 (5)	C48—C49—H49	118.9
C5—C4—H4	120.0	C49—C50—C51	119.4 (4)
C3—C4—H4	120.0	C49—C50—H50	120.3
C4—C5—C6	122.2 (5)	C51—C50—H50	120.3
C4—C5—H5	118.9	C52—C51—C50	118.6 (4)
C6—C5—H5	118.9	C52—C51—C54	118.4 (5)
C5—C6—C7	118.6 (4)	C50—C51—C54	123.0 (5)
C5—C6—C9	120.1 (5)	C51—C52—C53	121.3 (5)
C7—C6—C9	121.4 (5)	C51—C52—H52	119.4
C8—C7—C6	119.7 (5)	C53—C52—H52	119.4
C8—C7—H7	120.1	C52—C53—C48	120.2 (5)
C6—C7—H7	120.1	C52—C53—H53	119.9
C7—C8—C3	120.8 (5)	C48—C53—H53	119.9
C7—C8—H8	119.6	N8—C54—C51	124.5 (5)
C3—C8—H8	119.6	N8—C54—H54	117.8
N2—C9—C6	123.7 (5)	C51—C54—H54	117.8
N2—C9—H9	118.1	C56—C55—C60	117.6 (4)
C6—C9—H9	118.1	C56—C55—N8	119.9 (5)
C15—C10—C11	119.3 (5)	C60—C55—N8	122.4 (5)
C15—C10—N2	118.0 (5)	C55—C56—C57	122.6 (5)
C11—C10—N2	122.6 (5)	C55—C56—H56	118.7
C12—C11—C10	119.2 (5)	C57—C56—H56	118.7
C12—C11—H11	120.4	C56—C57—C58	119.4 (5)
C10—C11—H11	120.4	C56—C57—H57	120.3
C11—C12—C13	120.9 (5)	C58—C57—H57	120.3
C11—C12—H12	119.5	C59—C58—C57	118.6 (5)
C13—C12—H12	119.5	C59—C58—H58	120.7
C12—C13—C14	119.8 (5)	C57—C58—H58	120.7
C12—C13—H13	120.1	C58—C59—C60	122.0 (5)
C14—C13—H13	120.1	C58—C59—H59	119.0
C15—C14—C13	120.1 (5)	C60—C59—H59	119.0
C15—C14—H14	119.9	C59—C60—C55	119.6 (5)
C13—C14—H14	119.9	C59—C60—H60	120.2
C14—C15—C10	120.7 (5)	C55—C60—H60	120.2
C14—C15—H15	119.7	C62—N9—C63	128.8 (4)
C10—C15—H15	119.7	C62—N9—H9N	112 (3)
C17—N3—C18	129.1 (4)	C63—N9—H9N	119 (3)
C17—N3—H3N	119 (3)	C69—N10—C70	119.4 (5)
C18—N3—H3N	112 (3)	C62—C61—H61A	109.5
C24—N4—C25	118.2 (4)	C62—C61—H61B	109.5
C17—C16—H16A	109.5	H61A—C61—H61B	109.5
C17—C16—H16B	109.5	C62—C61—H61C	109.5
H16A—C16—H16B	109.5	H61A—C61—H61C	109.5
C17—C16—H16C	109.5	H61B—C61—H61C	109.5
H16A—C16—H16C	109.5	O5—C62—N9	122.7 (5)
H16B—C16—H16C	109.5	O5—C62—C61	122.2 (5)
O2—C17—N3	122.9 (4)	N9—C62—C61	115.1 (4)
O2—C17—C16	122.9 (5)	C64—C63—C68	119.2 (4)
N3—C17—C16	114.1 (4)	C64—C63—N9	125.3 (5)
N3—C18—C19	118.4 (4)	C68—C63—N9	115.5 (5)
N3—C18—C23	123.0 (4)	C63—C64—C65	118.8 (5)
C19—C18—C23	118.6 (4)	C63—C64—H64	120.6
C20—C19—C18	121.0 (5)	C65—C64—H64	120.6
C20—C19—H19	119.5	C66—C65—C64	122.9 (5)
C18—C19—H19	119.5	C66—C65—H65	118.6
C19—C20—C21	121.3 (5)	C64—C65—H65	118.6
C19—C20—H20	119.4	C65—C66—C67	117.1 (4)
C21—C20—H20	119.4	C65—C66—C69	122.9 (5)
C22—C21—C20	117.4 (4)	C67—C66—C69	120.0 (5)
C22—C21—C24	119.5 (5)	C68—C67—C66	121.4 (5)
C20—C21—C24	123.1 (5)	C68—C67—H67	119.3
C21—C22—C23	122.9 (5)	C66—C67—H67	119.3
C21—C22—H22	118.5	C67—C68—C63	120.6 (5)
C23—C22—H22	118.5	C67—C68—H68	119.7
C22—C23—C18	118.8 (5)	C63—C68—H68	119.7
C22—C23—H23	120.6	N10—C69—C66	122.9 (5)
C18—C23—H23	120.6	N10—C69—H69	118.5
N4—C24—C21	124.4 (5)	C66—C69—H69	118.5
N4—C24—H24	117.8	C75—C70—C71	119.0 (5)
C21—C24—H24	117.8	C75—C70—N10	117.0 (5)
C26—C25—C30	120.5 (5)	C71—C70—N10	123.9 (5)
C26—C25—N4	117.3 (5)	C72—C71—C70	120.3 (5)
C30—C25—N4	121.9 (5)	C72—C71—H71	119.9
C27—C26—C25	119.5 (5)	C70—C71—H71	119.9
C27—C26—H26	120.2	C71—C72—C73	120.3 (5)
C25—C26—H26	120.2	C71—C72—H72	119.9
C26—C27—C28	120.5 (5)	C73—C72—H72	119.9
C26—C27—H27	119.7	C74—C73—C72	119.6 (5)
C28—C27—H27	119.7	C74—C73—H73	120.2
C29—C28—C27	119.7 (5)	C72—C73—H73	120.2
C29—C28—H28	120.1	C75—C74—C73	120.3 (5)
C27—C28—H28	120.1	C75—C74—H74	119.9
C28—C29—C30	120.6 (5)	C73—C74—H74	119.9
C28—C29—H29	119.7	C74—C75—C70	120.6 (5)
C30—C29—H29	119.7	C74—C75—H75	119.7
C25—C30—C29	119.1 (5)	C70—C75—H75	119.7
C25—C30—H30	120.4	C77—N11—C78	128.0 (4)
C29—C30—H30	120.4	C77—N11—H11N	115 (3)
C32—N5—C33	130.9 (4)	C78—N11—H11N	117 (3)
C32—N5—H5N	115 (3)	C84—N12—C85	119.5 (5)
C33—N5—H5N	113 (3)	C77—C76—H76A	109.5
C39—N6—C40	119.6 (4)	C77—C76—H76B	109.5
C32—C31—H31A	109.5	H76A—C76—H76B	109.5
C32—C31—H31B	109.5	C77—C76—H76C	109.5
H31A—C31—H31B	109.5	H76A—C76—H76C	109.5
C32—C31—H31C	109.5	H76B—C76—H76C	109.5
H31A—C31—H31C	109.5	O6—C77—N11	125.5 (5)
H31B—C31—H31C	109.5	O6—C77—C76	120.4 (5)
O3—C32—N5	121.9 (5)	N11—C77—C76	114.0 (4)
O3—C32—C31	122.5 (5)	C83—C78—N11	115.8 (5)
N5—C32—C31	115.6 (4)	C83—C78—C79	120.2 (5)
C38—C33—N5	123.4 (5)	N11—C78—C79	124.0 (5)
C38—C33—C34	118.8 (5)	C80—C79—C78	117.9 (5)
N5—C33—C34	117.7 (4)	C80—C79—H79	121.1
C35—C34—C33	120.1 (5)	C78—C79—H79	121.1
C35—C34—H34	120.0	C79—C80—C81	122.9 (5)
C33—C34—H34	120.0	C79—C80—H80	118.6
C34—C35—C36	122.5 (5)	C81—C80—H80	118.6
C34—C35—H35	118.8	C80—C81—C82	117.8 (5)
C36—C35—H35	118.8	C80—C81—C84	122.0 (5)
C35—C36—C37	117.8 (4)	C82—C81—C84	120.2 (5)
C35—C36—C39	124.0 (5)	C83—C82—C81	120.0 (5)
C37—C36—C39	118.2 (5)	C83—C82—H82	120.0
C38—C37—C36	120.4 (5)	C81—C82—H82	120.0
C38—C37—H37	119.8	C82—C83—C78	121.1 (5)
C36—C37—H37	119.8	C82—C83—H83	119.4
C37—C38—C33	120.4 (5)	C78—C83—H83	119.4
C37—C38—H38	119.8	N12—C84—C81	122.1 (5)
C33—C38—H38	119.8	N12—C84—H84	119.0
N6—C39—C36	122.7 (5)	C81—C84—H84	119.0
N6—C39—H39	118.6	C86—C85—C90	118.3 (5)
C36—C39—H39	118.6	C86—C85—N12	123.3 (5)
C45—C40—C41	118.2 (5)	C90—C85—N12	118.2 (5)
C45—C40—N6	124.5 (5)	C87—C86—C85	120.8 (5)
C41—C40—N6	117.2 (5)	C87—C86—H86	119.6
C42—C41—C40	120.9 (5)	C85—C86—H86	119.6
C42—C41—H41	119.6	C86—C87—C88	120.3 (5)
C40—C41—H41	119.6	C86—C87—H87	119.8
C41—C42—C43	120.2 (5)	C88—C87—H87	119.8
C41—C42—H42	119.9	C87—C88—C89	119.7 (5)
C43—C42—H42	119.9	C87—C88—H88	120.2
C42—C43—C44	119.3 (5)	C89—C88—H88	120.2
C42—C43—H43	120.3	C90—C89—C88	120.1 (5)
C44—C43—H43	120.3	C90—C89—H89	120.0
C45—C44—C43	120.3 (5)	C88—C89—H89	120.0
C45—C44—H44	119.8	C89—C90—C85	120.8 (5)
C43—C44—H44	119.8	C89—C90—H90	119.6
C44—C45—C40	121.1 (5)	C85—C90—H90	119.6
C44—C45—H45	119.5	H1O—O1S—H2O	114 (4)
C40—C45—H45	119.5	H3O—O2S—H4O	97 (4)
C47—N7—C48	127.3 (4)	H5O—O3S—H6O	102 (4)
C47—N7—H7N	117 (3)	H7O—O4S—H8O	100 (4)
			
C3—N1—C2—O1	3.4 (8)	C48—N7—C47—O4	4.7 (8)
C3—N1—C2—C1	−175.3 (4)	C48—N7—C47—C46	−176.3 (4)
C2—N1—C3—C4	3.3 (8)	C47—N7—C48—C53	13.3 (8)
C2—N1—C3—C8	179.8 (4)	C47—N7—C48—C49	−169.6 (4)
C8—C3—C4—C5	2.1 (7)	C53—C48—C49—C50	−3.6 (7)
N1—C3—C4—C5	178.5 (5)	N7—C48—C49—C50	179.2 (4)
C3—C4—C5—C6	−0.4 (8)	C48—C49—C50—C51	0.3 (7)
C4—C5—C6—C7	−0.8 (8)	C49—C50—C51—C52	2.9 (7)
C4—C5—C6—C9	179.2 (5)	C49—C50—C51—C54	−179.0 (4)
C5—C6—C7—C8	0.2 (7)	C50—C51—C52—C53	−2.8 (7)
C9—C6—C7—C8	−179.8 (4)	C54—C51—C52—C53	178.9 (5)
C6—C7—C8—C3	1.6 (7)	C51—C52—C53—C48	−0.4 (8)
C4—C3—C8—C7	−2.7 (7)	C49—C48—C53—C52	3.6 (7)
N1—C3—C8—C7	−179.5 (4)	N7—C48—C53—C52	−179.3 (4)
C10—N2—C9—C6	177.3 (5)	C55—N8—C54—C51	177.2 (5)
C5—C6—C9—N2	179.9 (5)	C52—C51—C54—N8	178.1 (5)
C7—C6—C9—N2	−0.1 (8)	C50—C51—C54—N8	−0.1 (8)
C9—N2—C10—C15	141.4 (5)	C54—N8—C55—C56	136.3 (5)
C9—N2—C10—C11	−42.8 (7)	C54—N8—C55—C60	−46.3 (7)
C15—C10—C11—C12	0.2 (7)	C60—C55—C56—C57	0.5 (7)
N2—C10—C11—C12	−175.6 (5)	N8—C55—C56—C57	178.0 (5)
C10—C11—C12—C13	−0.1 (7)	C55—C56—C57—C58	−1.8 (8)
C11—C12—C13—C14	−0.6 (8)	C56—C57—C58—C59	1.1 (7)
C12—C13—C14—C15	1.2 (8)	C57—C58—C59—C60	1.0 (7)
C13—C14—C15—C10	−1.1 (8)	C58—C59—C60—C55	−2.4 (7)
C11—C10—C15—C14	0.4 (7)	C56—C55—C60—C59	1.6 (7)
N2—C10—C15—C14	176.3 (5)	N8—C55—C60—C59	−175.8 (5)
C18—N3—C17—O2	−3.6 (8)	C63—N9—C62—O5	−3.4 (8)
C18—N3—C17—C16	174.1 (5)	C63—N9—C62—C61	175.7 (4)
C17—N3—C18—C19	166.2 (5)	C62—N9—C63—C64	17.4 (8)
C17—N3—C18—C23	−13.3 (8)	C62—N9—C63—C68	−164.6 (4)
N3—C18—C19—C20	−178.6 (4)	C68—C63—C64—C65	0.5 (7)
C23—C18—C19—C20	0.9 (7)	N9—C63—C64—C65	178.4 (5)
C18—C19—C20—C21	1.2 (7)	C63—C64—C65—C66	−1.4 (8)
C19—C20—C21—C22	−2.2 (7)	C64—C65—C66—C67	1.3 (7)
C19—C20—C21—C24	177.7 (4)	C64—C65—C66—C69	−178.1 (5)
C20—C21—C22—C23	1.1 (7)	C65—C66—C67—C68	−0.5 (7)
C24—C21—C22—C23	−178.8 (5)	C69—C66—C67—C68	179.0 (5)
C21—C22—C23—C18	1.0 (8)	C66—C67—C68—C63	−0.3 (7)
N3—C18—C23—C22	177.5 (4)	C64—C63—C68—C67	0.3 (7)
C19—C18—C23—C22	−2.0 (7)	N9—C63—C68—C67	−177.8 (4)
C25—N4—C24—C21	−176.9 (5)	C70—N10—C69—C66	176.9 (5)
C22—C21—C24—N4	−175.6 (5)	C65—C66—C69—N10	169.5 (5)
C20—C21—C24—N4	4.6 (8)	C67—C66—C69—N10	−9.9 (8)
C24—N4—C25—C26	−136.0 (5)	C69—N10—C70—C75	140.5 (5)
C24—N4—C25—C30	48.7 (7)	C69—N10—C70—C71	−43.5 (7)
C30—C25—C26—C27	−0.8 (7)	C75—C70—C71—C72	−0.7 (8)
N4—C25—C26—C27	−176.1 (4)	N10—C70—C71—C72	−176.7 (5)
C25—C26—C27—C28	0.8 (8)	C70—C71—C72—C73	−0.8 (8)
C26—C27—C28—C29	−0.5 (8)	C71—C72—C73—C74	0.9 (8)
C27—C28—C29—C30	0.2 (8)	C72—C73—C74—C75	0.6 (8)
C26—C25—C30—C29	0.5 (7)	C73—C74—C75—C70	−2.2 (8)
N4—C25—C30—C29	175.6 (4)	C71—C70—C75—C74	2.2 (8)
C28—C29—C30—C25	−0.2 (7)	N10—C70—C75—C74	178.5 (5)
C33—N5—C32—O3	−5.6 (8)	C78—N11—C77—O6	1.1 (8)
C33—N5—C32—C31	174.5 (5)	C78—N11—C77—C76	−180.0 (4)
C32—N5—C33—C38	4.7 (8)	C77—N11—C78—C83	165.0 (5)
C32—N5—C33—C34	−178.3 (5)	C77—N11—C78—C79	−16.0 (8)
C38—C33—C34—C35	−0.3 (7)	C83—C78—C79—C80	−0.5 (7)
N5—C33—C34—C35	−177.5 (4)	N11—C78—C79—C80	−179.5 (5)
C33—C34—C35—C36	0.4 (7)	C78—C79—C80—C81	2.1 (7)
C34—C35—C36—C37	−0.4 (7)	C79—C80—C81—C82	−2.2 (7)
C34—C35—C36—C39	179.5 (5)	C79—C80—C81—C84	178.0 (5)
C35—C36—C37—C38	0.2 (7)	C80—C81—C82—C83	0.7 (7)
C39—C36—C37—C38	−179.6 (4)	C84—C81—C82—C83	−179.5 (4)
C36—C37—C38—C33	−0.2 (7)	C81—C82—C83—C78	0.8 (7)
N5—C33—C38—C37	177.2 (5)	N11—C78—C83—C82	178.2 (4)
C34—C33—C38—C37	0.2 (7)	C79—C78—C83—C82	−0.9 (7)
C40—N6—C39—C36	−178.1 (5)	C85—N12—C84—C81	−177.8 (5)
C35—C36—C39—N6	1.7 (8)	C80—C81—C84—N12	−172.3 (5)
C37—C36—C39—N6	−178.5 (4)	C82—C81—C84—N12	7.9 (7)
C39—N6—C40—C45	42.9 (7)	C84—N12—C85—C86	40.9 (7)
C39—N6—C40—C41	−140.8 (5)	C84—N12—C85—C90	−143.9 (5)
C45—C40—C41—C42	−1.2 (7)	C90—C85—C86—C87	1.3 (7)
N6—C40—C41—C42	−177.7 (4)	N12—C85—C86—C87	176.5 (5)
C40—C41—C42—C43	1.7 (8)	C85—C86—C87—C88	0.5 (8)
C41—C42—C43—C44	−1.6 (8)	C86—C87—C88—C89	−1.2 (8)
C42—C43—C44—C45	1.1 (7)	C87—C88—C89—C90	−0.1 (8)
C43—C44—C45—C40	−0.7 (7)	C88—C89—C90—C85	1.9 (8)
C41—C40—C45—C44	0.7 (7)	C86—C85—C90—C89	−2.5 (8)
N6—C40—C45—C44	176.9 (5)	N12—C85—C90—C89	−178.0 (5)

### Hydrogen-bond geometry (Å, °)

**Table:**

*D*—H···*A*	*D*—H	H···*A*	*D*···*A*	*D*—H···*A*
N1—H1N···O1S	0.88 (2)	2.00 (2)	2.872 (5)	173 (4)
N3—H3N···O2S	0.87 (2)	1.99 (2)	2.859 (5)	173 (4)
N5—H5N···O4S	0.86 (2)	2.09 (2)	2.919 (5)	163 (4)
N7—H7N···O3S	0.86 (2)	2.03 (2)	2.858 (5)	161 (4)
N9—H9N···O4	0.87 (2)	2.10 (2)	2.929 (5)	158 (4)
N11—H11N···O2	0.87 (2)	2.16 (3)	2.947 (5)	151 (4)
O1S—H1O···N4	0.83 (2)	2.29 (3)	2.939 (5)	136 (4)
O1S—H2O···O5	0.88 (2)	1.88 (2)	2.764 (5)	173 (4)
O2S—H3O···N2	0.85 (2)	2.16 (2)	3.006 (5)	177 (4)
O2S—H4O···O3	0.86 (2)	1.96 (2)	2.781 (5)	160 (5)
O3S—H5O···N6^i^	0.85 (2)	2.33 (3)	3.001 (5)	136 (3)
O3S—H6O···O1^i^	0.89 (2)	1.90 (2)	2.778 (4)	172 (4)
O4S—H7O···N8^ii^	0.87 (2)	2.09 (2)	2.954 (5)	171 (4)
O4S—H8O···O6^ii^	0.98 (2)	1.76 (2)	2.726 (5)	170 (4)

Symmetry codes: (i) *x*, *y*+1, *z*; (ii) *x*, *y*−1, *z*.

### Table 2 Dihedral angles (°) between the aromatic rings in each molecule of (I)

**Table:**

Plane–plane	Dihedral angle
*P*1–*P*2	42.4 (2)
*P*3–*P*4	52.8 (2)
*P*5–*P*6	43.7 (2)
*P*7–*P*8	48.0 (2)
*P*9–*P*10	53.8 (2)
*P*11–*P*12	48.1 (2)

Planes *P*1–*P*12 are those through the C3–C9, C10–C15, C18–C23, C25–C30, C33–C38, C40–C45, C48–C53, C55–C60, C63–C68, C70–C75, C78–C83 and C85-C90 rings, respectively.

### Table 3 Intermolecular distances (Å) of the atoms of the carbonyl groups to the aromatic ring between the paired molecules

**Table:**

Atom···plane	Distances
O1···*P*5, C2···*P*5	3.375 (4), 3.427 (6)
O2···*P*9, C17···*P*9	3.061 (4), 3.191 (6)
O3···*P*1, C32···*P*1	3.421 (4), 3.492 (6)
O4···*P*11, C47···*P*11	3.073 (4), 3.236 (6)
O5···*P*3, C62···*P*3	3.002 (4), 3.448 (6)
O6···*P*7, C77···*P*7	3.036 (4), 3.470 (6)

Planes *P*1, *P*3, *P*5, *P*7, *P*9 and *P*11 are those through the C3–C9, C18–C23, C33–C38, C48–C53, C63–C68 and C78–C83 rings, respectively.
